# *In Vitro* Micropropagation of *Aloe adigratana* Reynolds Using Offshoot Cuttings

**DOI:** 10.1155/2020/9645316

**Published:** 2020-04-16

**Authors:** Mulu Niguse, Desta Berhe Sbhatu, Haftom Baraki Abraha

**Affiliations:** ^1^College of Natural and Computational Science, Mekelle University, P.O. Box 231, Mekelle, Ethiopia; ^2^Mekelle Institute of Technology, Mekelle University, P.O. Box 1632, Mekelle, Ethiopia

## Abstract

This study aimed at developing a suitable and reproducible protocol for *in vitro* micropropagation of *Aloe adigratana* Reynolds using explants from offshoots with the help of the most commonly used plant growth regulators (PGRs). Explants were initiated in full-strength Murashige and Skoog (MS) media enriched with 0.2 mg/L benzylaminopurine (BAP) + 0.2 mg/L naphthaleneacetic acid (NAA). Shooting experiment was conducted in full-strength MS media enriched with 0/0, 0.5/0.5, 1.0/0.5, 1.5/0.5, and 2.0/0.5 mg/L BAP/NAA. Likewise, rooting experiment was carried out in half-strength MS media enriched with NAA at 0.5, 1.0, and 1.5 mg/L and indol-3-butyric acid (IBA) at 0.5, 1.0, and 1.5 mg/L. Finally, acclimatization study was conducted in greenhouse, nursery, and open field. The shooting response of the plant was best in MS media enriched with 1.0 mg/L BAP + 0.5 mg/L NAA. This media formulation resulted in the shortest mean number of days to shooting (14.00 ± 2.30 days), the highest mean shoot number (4.00 ± 3.40), and the second highest mean shoot length (8.60 ± 2.10 cm). IBA enhanced rooting at higher concentrations (1.0 and 1.5 mg/L), but NAA did the same at lower concentrations (0.5 and 1.0 mg/L). All plantlets (*n* = 62) survived primary acclimatization. Secondary acclimatization in composted and matured soil media under nursery and open field (sun light) condition produced 88 to 93% survival rate. The death of plantlets in the secondary acclimatization is accounted to mechanical damage. This study demonstrated that the tested PGRs were useful in enhancing the *in vitro* micropropagation of the plant. Future studies need to focus on optimizing the protocol for large-scale, commercial micropropagation.

## 1. Introduction

*Aloe* L. (Aloaceae) species are succulent flowering plants, highly valued in many communities of the world. The ethnobotanic, ethnomedicinal, and ethnoveterinary values of aloes are well-documented. Moreover, many *Aloe* species are extensively used in the preparation of cosmetic and toiletry industries. Aloes grow nearly in all parts of the world [1–4]. Different accounts put the number of *Aloe* species between 450 and 600. Ethiopia and Eritrea are believed to be home to 50 species of *Aloe* where 31 are endemic [3, 5–7]. Ethiopian and Eritrean aloes grow in various soil types and altitudes stretching from the sea level at Massawa (*Aloe eumassawana* Carter, Gilbert & Sebsebe) to ca. 3,500 meters at Ankober (*A. ankoberensis* Gilbert & Sebsebe) exhibiting a high degree of endemism. Many of them are restricted to very small geographic areas and ecological zones [3, 6].

Aloes have been used for a variety of purposes in ancestral and modern societies. They are used for preparing food, feed, and beverages, formulating ethnomedicinal and ethnoveterinary remedies and preparing traditional and modern cosmetic products. They are also grown for ornamental purposes. Aloe gels and latexes are used in treating bacterial, fungal, and viral diseases, healing wounds and skin burns, treating protozoan and helminthic infections, and normalizing noninfectious physiological ailments. Aloe-based cosmetic and healthcare products such as shampoos, moisturizers, and skin conditioners are good sources of revenues in many countries [1, 8–13].

The demand for aloes in the medicinal and cosmetic industries is increasing at an alarming rate, but large-scale production schemes to meet the demand are limited [1, 14]. Likewise, there is a growing interest to use Ethiopian aloes, such as *Aloe debrana* and *A. trichosantha*, for industrial inputs. Unfortunately, nearly all Ethiopian aloes are listed in the CITES (Convention on the International Trade in Endangered Species of Wild Fauna and Flora) Appendix II, implying that they cannot be exploited for commercial purposes from wild stand. Moreover, the natural propagation of aloes is very poor for some critical reasons. Vegetative propagation by lateral buds and offshoot is also very slow. Besides, sexual reproduction by seeds is ineffective due to male sterility and genetic unpredictability [6].

*Aloe adigratana* Reynolds is an endangered Ethiopian aloe restricted to the Tigrai floristic region. The species is hardy and massive, growing successfully in a diversity of habitats between 2,000 and 2,700 meters above sea level [6]. The plant has good potential to be exploited for commercial purposes. But its natural regeneration and *in vitro* propagation capacities are not explored. Thus, efforts towards rapid *in vitro* propagation of the species are needed. This study was conducted to develop a reproducible protocol of *in vitro* micropropagation of *A. adigratana* using the most common plant growth regulators (PGRs). The findings with the most common PGRs will help us in making further research for developing the best *in vitro* micropropagation protocol for the plant.

## 2. Materials and Methods

### 2.1. Location of Research Site

The experiment was conducted at the Tigrai Biotechnology Center Pvt. Ltd. Co. Lab, formerly known as Mekelle Plant Tissue Culture Laboratory. The center is located in Mekelle, Tigrai, Ethiopia (alt.: 1979 masl; lat.: 13° 30″0′ N; long.: 39° 28″11′ E), about 200 km southeast of the historic city of Aksum. The center has the capacity of producing more than 40 million plantlets per annum.

### 2.2. Collection and Preparation Explants

Many offshoots of *A. adigratana* Reynolds were collected from the south side of Mount Messebo, about seven kilometers north of the city center of Mekelle, Tigrai. Collection of biological materials by Ethiopian researchers for research is granted by Article 15, Clause 1, of the Access to Genetic Resources and Community Knowledge and Community Rights Proclamation of Ethiopia (No. 482/2006). Healthy offshoots with no symptoms of diseases were identified. Offshoots were dug out carefully and gently from the bases of mother plants with minimum mechanical damage and contamination. The external leaves were removed, and the shoots were trimmed to 1.5 to 2 cm long explants (*n* = 30).

### 2.3. Sterilization of Explants

Explants were sterilized sequentially by washing with running tap water for 30 minutes; dipping and stirring in a mixture of ridomile, kocide, and baylaton (1 : 1:1 v/v) for 10 minutes; dipping and stirring in Tween 20 solution for 10 minutes; rinsing with tap water four times; rinsing with distilled water two times to remove traces of detergent; dipping in 70% ethanol for 30 seconds; dipping and stirring in 20% sodium hypochlorite for 10 minutes; and rinsing with distilled water three times. Then, they were further sterilized in a laminar air flow cabinet by surface sterilization using freshly prepared HgCl_2_ (0.25% w/v aqueous solution) for 5 minutes followed by thorough washing using sterile distilled water to remove traces of HgCl_2_ [15–19].

### 2.4. Preparation of Propagation Media

Growth media were prepared as per the standard protocol of Murashige and Skoog (MS) [20] media by supplementing with PGRs, namely, benzylaminopurine (BAP), indol-3-butyric acid (IBA), and naphthaleneacetic acid (NAA). Full-strength (for initiation and shooting) and half-strength (for rooting) MS media were prepared by adding appropriate stock solutions of micronutrients, macronutrients, and additives and by enriching them with 30 g sucrose (C-source) and 8 g agar as a solidifying agent. The pH of the solutions was adjusted to about 5.8 by adding drops of 1 N HCl and 1 N NaOH as appropriate.

The initiation, shooting, and rooting media were prepared in 300 mL labeled magenta culture bottles by adding different concentrations and combinations of BAP, IBA, and NAA. The shoot initiation medium was composed of full-strength MS media enriched with 0.20 mg/L BAP and 0.20 mg/L. Likewise, shooting media were prepared in five treatments: one control without PGRs and four supplemented treatments with 0.5, 1.0, 1.5, and 2.0 mg/L of BAP plus constant (0.5 mg/L) concentration of NAA. Finally, rooting media were prepared in seven treatments: one control without PGRs and six enriched treatments with 0.5, 1.0, and 1.5 mg/L NAA and 0.5, 1.0, and 1.5 mg/L IBA. All treatments were replicated five times. Then, all the media were autoclaved at 121°C and 15 psi for 20 min and allowed to cool at room temperature to about 60°C. Finally, they were kept for a week to inspect their sterility before they are used.

### 2.5. Micropropagation Experiments

Shoot initiation was tested in full-strength MS media enriched with 0.20 mg/L BAP and 0.20 mg/L NAA to produce healthy and live (initiated) explants. Thirty (30) sterilized explants (1.5–2.0 cm long) were inoculated into 300 mL magenta culture bottles. One explant was inoculated into each magenta culture bottle. Then, they were incubated for 10 days in a growth room adjusted to 25 ± 0.5°C under fluorescent tube light with 16 hours photoperiod and 2000–2500 lux light intensity.

Shooting experiment was conducted in five treatments with five replications. Twenty-five (25) clean and initiated explants were aseptically transferred from the initiation media into sterile culture bottles containing 40 mL full-strength MS media enriched with PGRs. Each replication of the treatments was inoculated with one explant under laminar air flow cabinet. The explants were incubated by randomly placing them in growth room racks for four weeks with the same temperature and lighting conditions used in the initiation. Finally, shooting performance data were collected, and the shoots were transferred into fresh media and kept for additional four weeks to produce additional 2-3 cm long shoots.

Rooting experiment was conducted in seven treatments with five replications. Seventy (70) shoots (2-3 cm long) were collected from the shooting media and transferred to magenta culture bottles containing half-strength MS rooting media. Each replication of the treatments was inoculated with one shoot under the laminar air flow cabinet. The inoculated shoots were incubated by randomly placing them in growth room racks for four weeks with the temperature and lighting conditions described above to produce 3–5 cm long rooted shoots (i.e., plantlets) that are ready for acclimatization.

### 2.6. Acclimatization Experiments

Vigorous and same-sized plantlets (*n* = 62) were harvested from the rooting culture bottles and prepared for acclimatization experiment. The plantlets were carefully washed with hot water to remove traces of agar and sucrose that can prevent absorption of nutrients from the acclimatization media or substrates by the roots and become good media for the growth of contaminants. The plantlets were subjected to primary acclimatization in polycarbonate greenhouse. The acclimatization medium was coco peat in a pro-tray. The plantlets were kept in the greenhouse (relative humidity: 80 to 90%; temperature: 25 ± 2°C) for 14 days. Then, they were kept for additional 14 days by lowering the relative humidity to 60 to 70%. The plantlets were watered using a sprinkler to maintain the required humidity. Then, acclimatized, healthy, and same-sized plantlets were collected and distributed into four groups to be subjected to secondary acclimatization under two rooting media and two lighting conditions. The two rooting media were composted soil (i.e., sand, soil, and compost) and manured soil (i.e., sand, soil, and manure) at 1 : 1 : 1 ratio. The two light conditions were nursery shade and direct sunlight. The soil media were bagged in polyethylene bags (height 15 cm; dia. 9 cm). Then, the two groups of plantlets were planted in the composted soil, while the other two were planted in the manured soil. Afterwards, one group of plantlets in each soil medium was placed under nursery shade, and the second group of plantlets was placed in direct sunlight. The plantlets were watered daily with no fertilizer and studied for four weeks.

### 2.7. Data Collection and Analyses

Number (percentage) of explants initiated, number of days to shooting and rooting, number of shoots per explant, length of shoots and roots, number of roots per plantlet, and survival rates of plantlets to acclimatization were the sources of quantitative data. Data of days to shooting and rooting were collected by inspecting the incubated explants and plantlets every two days. Data on the number and length of shoots and roots were collected after four weeks of culturing. Qualitative data related to coloration, cleanliness, and contamination of explants and shoots were also recorded. Data analyses were carried out using analysis of variance (ANOVA) (SAS computer software Version 9.1.3) followed by post hoc comparison of means using the least significant difference (LSD). All comparisons were made at a priori set significance level of *p* ≤ 0.05.

## 3. Results and Discussion

### 3.1. Initiation Responses

Shoot initiation experiment was aimed at producing healthy and live (initiated) explants. It was tested in full-strength MS media enriched with 0.20 mg/L BAP and 0.20 mg/L NAA. Thirty (30) explants were cultured for 10 days. This experiment resulted in 27 (90%) healthy and live (i.e., initiated) explants. *In vitro* micropropagation studies with many other *Aloe* species reported that shoot-yielding structures or microshoots are initiated within few day of culturing [11, 15, 21–27]. These researchers showed that application of various combinations and concentrations of BAP and auxins like IAA, IBA, and NAA help in formulating the best initiation media.

### 3.2. Shooting Responses

The shooting response of *A. adigratana* explants is expressed in terms of days to shooting, number of shoots per explant, and length of shoots. Explants cultured in the five treatments resulted in statistically significantly different mean days to shooting, mean number of shoots per explant, and mean length of shoots per treatment (*p* ≤ 0.05) (Table 1).

The formulations of the PGRs affected the days to shooting. Explants cultured on full-strength MS medium enriched with 1.0/0.5 and 1.5/0.5 mg/L BAP/NAA initiated shoot significantly quicker in 14.0 and 14.4 days, respectively. Both treatments resulted in significantly lower mean days to shooting compared to the treatments enriched with lower and higher BAP concentration. Similar results were reported in *A. elegans* [21], *A. trichosantha* [22], *A. percrassa* [15], and *A. vera* [27]. In all cases, 0.50/0.50 to 2.0/0.50 mg/L BAP/NAA resulted in shooting in 14 to 17 days. The lack or slow shooting in PGR-free MS media is observed by many researchers in several *Aloe* species [15, 19, 21, 22, 25–29].

**Figure 1:**
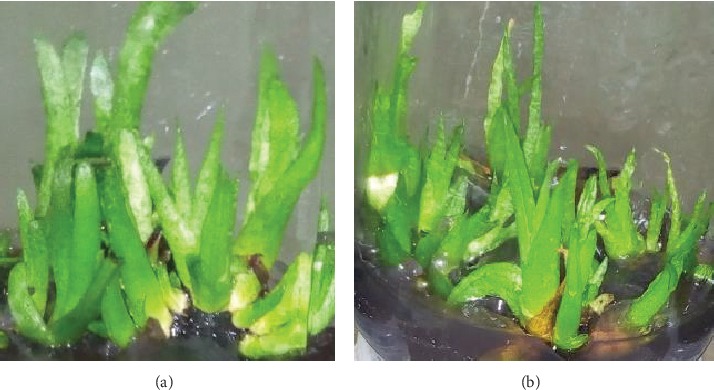
Four-week-old *A. adigratana* shoot cultured in MS media enriched with 1.0/0.5 mg/L BAP/NAA; (a) bottle #1; (b) bottle #2.

The use of combination of auxins and cytokinins with MS media is known to be effective for optimal *in vitro* shoot proliferation in many species including aloes as compared to the use of either group of PGRs [11, 25, 30, 31]. But establishing the best combinations and concentrations of auxins and cytokines for better shooting of explants is often tricky. The shooting response of *A. adigratana* explants in terms of mean shoot number per explant showed that 1.0/0.5 mg/L BAP/NAA is the best combination compared to the other combinations with lower and higher concentrations of BAP (*p* ≤ 0.05) (Table 1; Figure 1). Plantlets cultured in unsupplemented media were least responsive. Similar findings were reported in *A. percrassa*. Explants cultured in 1.0/0.5 mg/L BAP/NAA produced a higher mean shoot number (8.0) compared to those cultured in MS media enriched with lower and higher concentration of BAP [15].

Many researchers reported varied shooting responses with *A. vera*. For example, Daneshvar et al. [32] observed the highest shoot proliferation (28.47 shoots) with 2.5/0.15 mg/L BAP/NAA. Zakia et al. [33] recorded the highest shooting performance (11.18 shoots) with 0.5/0.5 mg/L BAP/NAA. Likewise, Kiran [34] observed a high mean shoot number (5) with 2.0/0.20 mg/L of BAP/NAA. Furthermore, other researchers observed 14 to 16 shoots per explant with 4.0/0.2 mg/L BAP/NAA in *A. vera* [25, 35]. This difference may be due to the type of explant and genotypic difference that often affect tissue culture response. Different genotypes respond differently to *in vitro* culture of shoots and roots [36].

The shooting response of the species, in terms of mean shoot length per explant, showed that nearly all treatments resulted in higher or comparable mean length of shoots. It was observed that all the treatments resulted in fewer but longer shoots. Therefore, 1.0/0.5 mg/L BAP/NAA as well resulted in better shooting response in terms of mean shoot length per treatment (8.6). Other researchers with Ethiopian aloes reported similar observations: e.g., Welehaweria [21] (mean shoot length of 9.2 cm), Hailu [22] (mean shoot length of 4.63 cm), and Abraha et al. [15] (mean shoot length of 5.2 cm).

Similar to the present study, one study with *A. vera* reported lower shooting response with increasing or decreasing BAP concentration from about 1.0 mg/L [37]. But many studies on *A. vera* reported better shooting responses (as a function of shoot length) in MS media enriched with 0.5 to 4.0 mg/L BAP in combination with 0.0 to 0.50 mg/L NAA or IBA [11, 19, 24–27, 33, 35, 38–40]. Higher concentration auxins (IAA, IBA, or NAA) up to 1.0 mg/L did not lead to better shooting response [27]. Generally, full-strength MS media enriched with 1.0/0.50 mg/L BAP, and NAA was found to be optimum for promoting shoot proliferations in *A. adigratana*. PGR supplementation was also observed to be necessary for shooting response in the plant.

### 3.3. Rooting Response

The rooting response of the *A. adigratana* was studied with 2-3 cm long shoots generated from the shooting experiment. Individual shoots were harvested by carefully excising from proliferated explant clumps. The rooting response is expressed in terms of days to rooting, number of roots per explant, and length of roots. Data analyses using ANOVA revealed that the rooting responses were affected by the types of PGRs and concentrations. Mean differences are statistically significant (*p* ≤ 0.05; Table 2).

It is well-established that auxins (IAA, IBA, and NAA) are efficient in facilitating rooting of aloes under *in vitro* conditions [13, 19, 26, 29, 41, 42]. Thus, many of such research studies focus on establishing the optimum concentrations for best responses. The present study showed that the shoots of *A. adigratana* cultured in unsupplemented MS media failed to root by the 30^th^ day. In case of the enriched treatments, IBA-enriched treatments resulted in quicker response in all concentrations, whereas IBA resulted in rooting in 15 to 18 days in all concentrations, and NAA yielded the same result at higher concentration (1.5 mg/L). Studies with shoots of *A. elegans* [21], *A. trichosantha* [22], *A. vera* [25], and *A. barbadensis* [23] reported the absence of rooting in nonenriched MS media.

IBA and NAA are commonly used in rooting of shoots in *A. vera.* Supplementation of half-strength MS media with 0.5 to 1.5 mg/L IBA and NAA was found to be the best in enhancing root formation in *A. vera* shoots [39]. The present study showed that IBA at 0.5 to 1.5 mg/L produces comparable results with regard to the mean number of roots. Even though lower concentration of IBA (0.5 mg/L) resulted in quicker rooting, increasing the concentration to 1.0 and 1.5 mg/L led to better rooting response in terms of root number and length. Similar result was reported in *A. elegans* [21].

Increasing the concentrations of NAA from 0.5 mg/L to 1.5 mg/L significantly lowered the mean number of days to rooting. However, the mean number and length of roots can be considered as the characteristics of good rooting response. NAA resulted in better effects in lower concentrations (0.5 and 1.0 mg/L) than in higher concentration (1.5 mg/L). Similar patterns were reported with *A. elegans* [21], *A. trichosantha* [22], *A. percrassa* [15], *A. barbadensis* [23], *A. vera* [32], and *A. polyphylla* [43]. Other workers also reported better rooting responses with NAA at 0.5 mg/L [26, 42, 44].

Table 2 shows that all the enriched treatments resulted in a mean root number between 2.46 and 5.62. Although the statistical differences among the treatments are not profound, IBA yielded better results in terms of mean root length. Similar findings were reported by many other researchers [13, 25, 32, 45]. On the other hand, the mean root length increased with increasing the concentration of IBA from 0.5 to 1.5 mg/L, while the reverse was true with NAA. In line with this, Abdi et al. [11] reported declining mean root length in *A. vera* with increasing the concentration of NAA.

### 3.4. Acclimatization of Plantlets

Rooted shoots (i.e., plantlets) were taken out of the rooting culture media after four weeks. Then, they were carefully washed by dipping in 40°C hot water for about 5 minutes to remove the agar residues and oily stuff off their roots. Finally, 62 healthy and vigorous plantlets (≥5 cm) were harvested for acclimatization. Acclimatization of the plantlets was carried out by moving them through a changing microclimate and planting media. The 62 plantlets were planted in a pro-tray filled with coco peat and were subjected to a four-week greenhouse primary acclimatization with changing microclimate from high relative humidity (RH), low lighting, and high temperature to low RH, high lighting, and high temperature. All (100%) of the plantlets have survived. Then, the plantlets were divided into four groups of 15, 15, 16, and 16. The first two groups were planted in composted soil media, and the second two groups were planted in manured soil media filled in polyethylene bags (height 15 cm; dia. 9 cm). One group planted in composted media and another one planted in the manured media were put under nursery shade while the other two groups were placed under direct sunlight for four weeks. The moisture content of the rooting media was maintained by sprinkling with distilled water. Secondary acclimatization has resulted in 88–93% survival (Table 3).

The survival of the plantlets during the secondary acclimatization was not affected by light conditions. The death of one plantlet from each group was suspected to be because of physical damage during transplanting. Some of the roots were delicate and can easily rot to cause the death of plantlets. Some plantlets put under the nursery shade have shown some symptoms of leaf tip necrosis, but their growth was not affected. Generally, *Aloe* species that survive primary acclimatization are highly likely to survive secondary acclimatization and thrive. Many researchers observed 85–100% survival rates in *A. vera* [11, 13, 19, 29, 30, 36, 45]. Moreover, similar observations were reported in other species such as *A. elegans* [21], *A. trichosantha* [22], and *A. percrassa* [15].

## 4. Concluding Remarks

*A. adigratana* is restricted to the Tigrai floristic region, Ethiopia [6]. Records in the Kew botanic garden claim that it also grows in Eritrea. Fortunately, the plant grows successfully in many parts of the Tigrai floristic region between 2,000 and 2,700 meters. It is planted as fences of backyards, farmlands, churchyards, footpaths, and area enclosures as well as demarcations of farmlands and other properties. The plant is also planted across gullies to hold soil erosion. However, the plant is being threatened by various local land use measures.

The natural regeneration capacity and commercial and ecological significance of the plant are not empirically explored. Like many species of *Aloe*, *A. adigratana* can be a good source of phytochemicals with medicinal, nutritional, and pharmaceutical potential. Exploring into the *in vitro* micropropagation of the plant is one effort in a large-scale project aiming at elucidating its regeneration, physicochemical, and agronomic characteristics. As the present study shows, the plant can easily and successfully be propagated *in vitro* with the help of the commonly used PGRs, namely, BAP, IBA, and NAA. The results of the study will serve as important foundation for future research using many different media formulations and combinations, refined concentrations of PGRs, and various sources of explants to develop an optimized protocol of large-scale *in vitro* micropropagation.

## Figures and Tables

**Table 1:** Shooting response of *A. adigratana* Reynolds in different concentrations of BAP and constant concentration of NAA.

PGRs (mg/L)		Mean no. of days to shooting	Mean no. of shoots per explants (*n*)	Mean length of shoots per treatment (cm)
BAP	NAA			
0.0	0.0	29.40 (0.90)^a^	0.80 (0.45)^b^	11.00 (3.50)^a^
0.5	0.5	23.40 (2.30)^b^	1.60 (0.55)^b^	7.80 (1.30)^bc^
1.0	0.5	14.00 (3.20)^c^	4.00 (3.40)^a^	8.60 (2.10)^ab^
1.5	0.5	14.40 (2.40)^c^	1.60 (0.90)^b^	5.60 (1.15)^c^
2.0	0.5	23.60 (3.40)^b^	1.00 (0.70)^b^	8.60 (1.15)^ab^
Mean		20.96	1.80	8.32
CV (%)		11.63	90.43	26.70
LSD		3.26	2.18	2.98

Means in the same column with different letters are statistically significantly different at *p* ≤ 0.05; CV: coefficient of variance (%); LSD: least significant difference.

**Table 2:** Rooting response of *A. adigratana* with different concentrations of NAA and IBA.

PGRs (mg/L)		Days to rooting	Mean no. of roots per explants (*n*)	Mean length of roots per explants (cm)
Control	0.0	—	—	—
IBA	0.5	14.60 (2.90)^d^	8.40 (3.40)^b^	3.86 (1.50)^ab^
	1.0	18.20 (2.60)^bc^	11.00 (3.75)^a^	5.10 (2.60)^a^
	1.5	15.00 (2.55)^cd^	9.40 (4.80)^b^	5.62 (3.10)^a^
NAA	0.5	24.20 (1.90)^a^	13.60 (6.95)^a^	4.04 (1.40)^ab^
	1.0	20.00 (3.50)^b^	11.80 (6.90)^a^	3.64 (0.80)^ab^
	1.5	15.00 (2.20)^cd^	10.40 (5.60)^ab^	2.46 (1.80)^b^
	Mean	19.57	9.23	3.53
	CV (%)	12.73	52.26	52.33
	LSD	3.25	6.29	2.41

Means with different letters in the same column are significantly different at *p* ≤ 0.05; CV coefficient of variance (%); LSD: least significant difference.

**Table 3:** Survival of plantlets of *A. adigratana* Reynolds to acclimatization.

Acclimatization media	Light condition	Quantity	Survival (%)
Artificial media (100% coco peat)	Greenhouse	62	100
Composted media (soil, sand, and compost at 1 : 1 : 1)	Nursery shade	15	93.3
	Direct sunlight	15	93.3
Manured media (soil, sand, and manure at 1 : 1 : 1)	Nursery shade	16	87.5
	Direct sunlight	16	87.5

## Data Availability

The datasets used and/or analyzed during the current study are available from the corresponding author on reasonable request.
